# Thoracoscopic staged repair for type A and type B esophageal atresia: outcomes from a tertiary center

**DOI:** 10.1007/s00383-026-06368-9

**Published:** 2026-03-04

**Authors:** Natalia Newland, Jiri Snajdauf, Jitka Styblova, Stepan Coufal, Tereza Bartosova, Michal Rygl, Alena Kokesova

**Affiliations:** 1 Department of Paediatric Surgery, 2nd Faculty of Medicine, Charles University in Prague and Motol University Hospital, Prague, Czech Republic; 2 Laboratory of Cellular and Molecular Immunology, Institute of Microbiology, Czech Academy of Sciences, Prague, Czech Republic

**Keywords:** Long-gap esophageal atresia, Gross type A and B, Thoracoscopy, Staged internal traction, Gastric interposition

## Abstract

**Purpose:**

To evaluate outcomes of thoracoscopic staged internal traction combined with a waiting period in long-gap esophageal atresia (LGEA) at a single center.

**Methods:**

Retrospective analysis of perinatal characteristics, surgical interventions, postoperative complications, and long-term outcomes in LGEA patients undergoing delayed thoracoscopic staged repair between 2018 and 2024.

**Results:**

Among141 esophageal atresia repairs, 15 patients had LGEA (13 Gross type A, 2 type B). All patients underwent gastrostomy placement. The first thoracoscopic stage occurred at a median age 2.4 months (1.1–3.7). Internal traction was applied in 13/15 (86%), two required gastric interposition (GI) for an extreme long gap diagnosed at initial thoracoscopy. Traction patients underwent a median of 2 tractions (1–3). Delayed anastomosis was achieved in 10/13 (77%) within a median 11.5 days (6–43) from first thoracoscopy. In three, anastomosis remained unfeasible after traction due to a persistent long gap, necessitating GI. Complications included one recurrent fistula, one leak, and five strictures requiring a median 1.5 dilatations (1–7). At a median 50-month follow-up (12–91), all delayed-anastomosis patients and 60% of GI patients tolerated full oral feeds.

**Conclusion:**

A delayed thoracoscopic staged internal traction enabled safe anastomosis with a low complication rate; however, one-third of patients required GI.

## Introduction

Long-gap esophageal atresia (LGEA) is known to be the most severe type of esophageal atresia. The distance between the proximal and distal esophageal pouches precludes a primary, tension-free anastomosis at the initial surgery, necessitating more advanced reconstructive strategies.

Owing to the inconsistent use of the LGEA definition, published data on the outcomes of different surgical approaches remain limited. For the purpose of this study, LGEA was defined as any esophageal atresia (EA) without intra-abdominal air, as has been endorsed by the International Network of Esophageal Atresia (INoEA) and the ERNICA expert groups [1, 2]. Patients with LGEA typically face a more complex clinical course, often requiring multiple surgeries to achieve esophageal continuity and exhibiting a higher risk of failure to preserve the native esophagus. Consequently, this comes with the expectations of increased complication rates, challenging peri-operative and long-term care and prolonged hospitalization. Given this complexity, the care of LGEA is rightfully recommended to be handled in a specialised centre with multidisciplinary expertise [2].

The optimal surgical strategy for managing LGEA remains a subject of ongoing debate. Most authors and guidelines agree that preservation of the native esophagus should be the primary focus of our efforts [1–4]. With advances in thoracoscopic techniques, the minimally invasive approach has naturally emerged as a promising alternative in the treatment of LGEA—whether through performing delayed primary anastomosis (DPA) [5, 6], aiding in external traction [7], or allowing for a fully minimally invasive internal traction strategy [8, 9]. Other questions remain unanswered, including the optimal timing of reconstructive surgeries, type of care during the waiting period and outcomes of different approaches.

In this study, we report the outcomes of a delayed thoracoscopic staged internal traction for type A and type B LGEA, based on our tertiary centre experience with national care centralization.

## Materials and methods

1. Study population

A retrospective longitudinal cohort study was conducted including all patients with esophageal atresia (EA) treated at our Pediatric Surgery Department between January 2018 and December 2024. Inclusion criteria were long-gap EA and an intended thoracoscopic staged internal traction repair as the initial operative approach. Long-gap EA was defined as EA without intra-abdominal air on the initial radiograph, consistent with Gross type A and B esophageal atresia.

Following institutional ethical committee approval (EK 544.1/223), electronic medical records of all patients were systematically reviewed. The extracted data included perinatal characteristics, associated anomalies, type of surgical procedure, number of surgeries required to achieve anastomosis, age at anastomosis, postoperative leakage, stricture, number of dilatations, need for future fundoplication, time to achieve full oral feeding, musculoskeletal deformities, and overall mortality.

2. Surgical details

As per institutional protocol for patients with esophageal atresia without intra-abdominal air, all patients underwent gastrostomy placement after birth. The first esophageal surgery was generally indicated at around two months of age, allowing time for spontaneous growth of the esophagus. During the waiting period, patients remained hospitalized and were managed with intermittent esophageal suction and bolus gastrostomy feeding. Prior to all surgeries, tracheoscopic evaluation was performed to rule out the presence of a proximal fistula.

Thoracoscopy was performed in the standard fashion in a semi prone position using a 5-mm endoscope and 3-mm instruments. In patients undergoing internal traction, both pouches were mobilised and approximated using sliding knots secured with clips, as described by Patkowski [10]. Traction procedures were repeated until a promising distance was achieved allowing for a delayed anastomosis. Individual stages were timed typically at weekly intervals, guided by the overall condition of the children. When anastomosis was not feasible, a cervical esophagostomy was fashioned. Parents were instructed in sham feeding, and patients were discharged home to await definitive replacement surgery. Gastric interposition was the chosen method of esophageal replacement and was generally performed at approximately 1.5 years of age, according to the technique described by Spitz [11].

3. Statistical analysis

Continuous variables were described as median and range. Categorical variables were described as absolute frequencies and percentages. Between-group comparisons for continuous data used the Mann–Whitney U test and *p* < 0.05 was considered statistically significant.

## Results

Over the 7-year period from 1 January 2018 to 31 December 2024 a total of 141 patients with esophageal atresia underwent surgical repair at our tertiary centre. Among these, 15 patients (10.6%) were diagnosed with EA without intra-abdominal air, including 13 with Gross type A and 2 with Gross type B. All patients met the inclusion criteria of this study.

Patient characteristics are summarized in Table 1. The cohort included 8 female and 7 male patients. The median birth weight was 2450 g (range 710–2970) and the median gestational age was 35 weeks (range 27–39). A statistically significant difference in birth weight was observed between patients who underwent successful delayed anastomosis and those requiring gastric interposition (median 2450 g vs. 1490 g, p value = 0.028). Antenatal suspicion of esophageal atresia was raised in 11 out of 13 pregnancies that were followed (85%), based on ultrasonographic findings of polyhydramnios and an absent or small gastric bubble. Associated anomalies were detected in 53% of patients with LGEA. The most common anomalies included cardiovascular (13%) and genitourinary (13%), followed by anorectal (6%), gastrointestinal (6%) and skeletal (6%).

**Table 1:** Patient characteristics

Variable	Value
EA cases during study period, N	141
LGEA, n/N (%)	15/141 (10.6%)
Gross type A, n/N (%)	13/15 (86.7%)
Gross type B, n/N (%)	2/15 (13.3%)
Male, n/N (%)	7/15 (46.7%)
Birth weight, g (median, range)	2450 (710–2970)
Gestational age, weeks (median, range)	35 (27–39)
Prenatal suspicion, n/N (%)	11/13 (84.6%)
Associated anomalies, n/N (%)	8/15 (53.3%)

Following postnatal stabilization and adaptation, all patients underwent gastrostomy placement in the first days of life. The first thoracoscopic esophageal surgery was performed at a median age of 2.4 months (range 1.1–3.7) and consisted of internal traction in 13 out of 15 patients (87%). In the remaining two patients, thoracoscopy revealed no identifiable lower or upper pouch. These patients subsequently underwent cervical esophagostomy and later gastric interposition (see Table 2).

**Table 2:** Operative strategy and timing

Variable	Value
*Initial procedure:*	
Gastrostomy placement after birth, n/N (%)	15/15 (100%)
*First esophageal surgery:*	
Thoracoscopic revision, n/N (%)	15/15 (100%)
Staged internal traction, n/N (%)	13/15 (86.7%)
Esophagostomy, n/N (%)	2/15 (13.3%)
*Final reconstruction*	
Delayed anastomosis, n/N (%)	10/15 (66.7%)
Gastric interposition (GI), n/N (%)	5/15 (33.3%)
*Timing*	
Age at first esophageal surgery, months (median, range)	2.4 (1.1–3.7)
Age at esophageal anastomosis, months (median, range)	2.9 (1.5–3.8)
Age at gastric interposition, months (median, range)	21 (13–29)

Among the 13 patients who underwent staged internal traction, the median number of traction procedures before anastomosis was achieved was 2 (range 1–3). Delayed anastomosis was successfully achieved in 10 out of 13 patients (77%) in a median 11.5 days (range 6–43) from first thoracoscopy. The remaining three patients (one with 2 and two with 3 traction attempts) did not achieve successful anastomosis. This was due to a persistent long gap in two cases and a long ischemic stricture precluding re-anastomosis in one case. When analysis was limited only to patients who achieved successful anastomosis, the median number of tractions was 1.5 (range 1–3).

In all, of the total 15 patients with LGEA, 10 patients (67%) achieved a successful delayed anastomosis at a median age of 2.9 months (range 1.5–3.8) using thoracoscopic staged internal traction. The remaining five patients (33%) required gastric interposition, which was performed at a median age of 21 months (range, 13–29 months).

Across the whole LGEA cohort, post-operative complications included one recurrent upper tracheo-esophageal fistula (TEF), which was successfully repaired via a cervical approach. One patient developed an anastomotic leak, which was managed conservatively. Anastomotic strictures occurred in five out of ten patients after delayed esophageal anastomosis and in none after gastric interposition. Strictures required a median of 1.5 dilatations (range 1–7) within the first year of age (see Table 3).

**Table 3:** Postoperative complications

Variable	Value
Recurrent upper tracheoesophageal fistula (TEF), n/N (%)	1/15 (6.7%)
Anastomotic leak, n/N (%)	1/15 (6.7%)
Anastomotic stricture (delayed anastomosis), n/N (%)	5/10 (50%)
Anastomotic stricture (gastric interposition), n/N (%)	0/5 (0%)
N. dilatations (first year of life), (median, range)	1.5 (1–7)
Fundoplication, n/N (%)	0/15 (0%)

Regarding long-term morbidity (summarized in Table 4), all patients with delayed anastomosis remained on proton pump inhibitor (PPI) prophylaxis. No patient required fundoplication for gastroesophageal reflux disease (GERD) during the follow-up period. Tracheomalacia was documented in 9 patients of the entire cohort (60%), though none required surgical treatment at the time of last follow-up. A musculoskeletal deformity developed in one patient who underwent gastric interposition via laparotomy following two thoracoscopies.

All patients who underwent delayed esophageal anastomosis achieved full oral feeding. The median time to reach full oral intake was 32 days (range 15–460) after anastomosis creation. Among patients with gastric interposition, 60% achieved full oral feeds and one patient died before reaching this stage.

The median follow-up time for patients with delayed anastomosis was 65 months (range 12–91) and for gastric interposition patients 31 months (range 19–73). There was one death resulting from aspiration at home in a gastric interposition patient.

**Table 4:** Long-term outcomes

Variable	Delayed anastomosis (*n* = 10)	Gastric interposition (*n* = 5)
Full oral feeds, n/N (%)	10/10 (100%)	3/5 (60%)
Time to full oral feeds after anastomosis, days (median, range)	32 (15–460)	–
PPI prophylaxis, n/N (%)	10/10 (100%)	0/5 (0%)
Tracheomalacia, n/N (%)	7/10 (70%)	2/5 (40%)
Musculoskeletal deformities, n/N (%)	0/10 (0%)	1/5 (20%)
Mortality, n/N (%)	0/10 (0%)	1/5 (20%)
Follow-up, months (median, range)	65 (12–91)	31 (19–73)

## Discussion

This single-centre study evaluated outcomes of a combined surgical strategy for patients with long-gap esophageal atresia (Gross type A and B). The surgical approach consisted of an initial waiting period to allow for esophageal and patient growth, followed by thoracoscopic staged internal traction repair. In our experience, this delayed staged approach was associated with a safe anastomosis, low rate of postoperative complications and enabled operating on clinically stable and larger infants.

According to the literature LGEA accounts for about 10% of all esophageal atresia cases, and most surgeons encounter fewer than one LGEA case per decade [2]. Consequently, LGEA is challenging not only from an operative technique standpoint, but also in perioperative and long-term care.

Multiple operative techniques have been described in the literature, with preservation of the native esophagus prioritized as the first line of treatment. Since the first thoracoscopic LGEA repair in 1999 [12] and with growing experience of surgeons in minimally invasive techniques, thoracoscopy has emerged as beneficial also in the treatment of LGEA. Nevertheless, the optimal surgical strategy and its timing remain of on-going discussions.

Within this ongoing debate, two principal strategies are currently pursued. Most authors advocate for a delayed primary anastomosis (DPA) allowing time for spontaneous growth of the esophagus before repair, performed open or thoracoscopically [1, 3, 5, 13, 14]. More recently, however, other authors favor earlier continuity using thoracoscopic elongation techniques via external or internal traction [7–9]. Although the latter bring a benefit of avoiding gastrostomy, it often entails multiple staged procedures in early neonatal life.

Accordingly, beyond the choice of technique, timing of surgeries is another arising question. We present outcomes of a combined approach that incorporates a waiting period for esophageal growth followed by thoracoscopic staged internal traction to maximize native esophageal length. Beyond facilitating esophageal growth, the possible other benefit of a waiting strategy is operating on a larger and clinically more stable infant which is especially advantageous in a technically challenging procedure such as LGEA repair. Secondly, LGEA often necessitates multiple thoracic procedures, with potential exposure to prolonged general anaesthesia or sedation between surgeries. Neonatal thoracic non-cardiac surgery has been associated with an increased risk of brain injury and subsequent neurodevelopmental delay [15–17]. A waiting period may shift these exposures out of the vulnerable neonatal window to a safer later period.

In our series, interestingly, despite employing a waiting period, we could not perform a delayed primary anastomosis (DPA) in any of our patients. This contrasts with other studies, where after the waiting period all or most patients underwent DPA [4, 5]. This likely reflects the team’s learning curve with thoracoscopy for LGEA, favoring a cautious approach to perform a safe anastomosis with less tension after traction period. Consistent with this approach, we observed only one postoperative leak in our series. Notably, Sakuov et al. also combined a 3–4 month waiting period before initiating traction stages and reported zero anastomotic leaks [18]. By contrast, studies favoring either DPA without prior traction or staged traction procedure in the early neonatal period without waiting period describe higher leak rates [4, 19, 20].

Although traction-based elongation techniques have yielded heterogeneous results across centres and some authors advise using it with great caution if at all [21, 22], our data suggest that esophageal growth with time alone might not be sufficient for a safe anastomosis in all patients. In our cohort, combining a waiting period with staged thoracoscopic traction was associated with no subsequent severe GERD requiring fundoplication. In contrast, in the series by Rothenberg and similarly by Zani et al., delayed primary anastomosis without traction led to fundoplication in nearly half of patients [4–6]. Likewise, a study on outcomes by van Serooskerken employing traction in early neonatal period without a waiting time reported fundoplication in 91% of cases [20]. Overall, our combined approach may capture advantages of both approaches while mitigating postoperative complications.

Gastric interposition was required in 33% of our LGEA patients. In two cases, this was an immediate decision owing to an extreme long-gap where no upper or lower pouch could be identified. The remaining three proceeded to gastric interposition after failure to achieve a successful delayed anastomosis despite staged internal traction. Comparable rates have been reported by other authors, such as 23% in the recent study by Celtik et al. [19] and 37% in centre B in a comparative study by Borselle et al. [23]. National surveys from the UK and the Nordic countries likewise report gastric interposition rates of 53% and 47.9%, respectively for Gross type A [24, 25]. In our series, infants who required gastric interposition had significantly lower birth weights than those with successful anastomosis, which may have contributed to a longer gap and increased tissue fragility.

Some surgeons advocate early repair to reduce the burden of prolonged hospitalization on the families [7, 26] and to potentially improve long-term functional outcomes of these patients. One of them is oral feeding and early restoration of esophageal continuity may mitigate later oral aversion [20]. In our series, the median time to full oral feeds was 32 days after anastomosis achievement; however the maximum was as long as 460 days. Furthermore, other authors publish even worse experience with only 18% of patients taking solids and liquids orally at a 30-day interval [13]. Notably, Tainaka et al. who published outcomes of a two-staged thoracoscopic repair and completed anastomosis relatively early (between 6 and 24 days of life), also experienced difficulties reaching oral feeding, which was on average 45 days after anastomosis [27]. This is a worrying outcome and underscores the importance of early proactive feeding rehabilitation, such as sham feeding, which is also recommended by ERNICA guidelines and supported by additional studies [1, 26, 28, 29] Furthermore, as the benefits of the waiting strategy mentioned above might be outweighed by the drawbacks of prolonged hospitalization, an appealing solution is the so-called Stockholm protocol [30, 31]. This approach delineates structured family involvement, beginning with sham feeding and, in selected cases, permits completion of the waiting period at home. A similar concept was previously proposed by Aziz et al. [32]. We contend that the success of any treatment strategy should be measured, in part, by its support for as normal early development as possible.

This single-centre study is limited primarily by its retrospective design, which constrained some data capture and precluded assessment of additional outcomes such as neurodevelopment. The small sample size and variable follow-up length further reduce statistical power and may under detect late complications. In addition, the absence of a control group precludes comparative or multivariable analyses. Accordingly, the results should be interpreted as hypothesis-generating and call for validation in larger, multicentre cohorts. Against these limitations, the study has several strengths. We examined a clearly defined LGEA cohort treated with a uniform thoracoscopic pathway (waiting period with staged internal traction), thereby reducing treatment heterogeneity. Concentration of cases within a centre with care centralization with a team dedicated to treatment and follow-up of EA patients also limits variability. These features support the relevance of our observations while underscoring the need for prospective, multicentre confirmation.

## Conclusion

This study presents the outcomes of a delayed thoracoscopic staged internal traction strategy for patients with long-gap esophageal atresia. The findings indicate that combining a waiting period for esophageal and patient growth with a minimally invasive thoracoscopic staged internal traction technique which permits maximal esophageal mobilization can facilitate a safe anastomosis. This approach appears to reduce post-operative complications, such as anastomotic leak and the subsequent need for fundoplication. All patients who underwent delayed anastomosis ultimately achieved full oral feeding, although this process might be accelerated through the incorporation of sham-feeding protocols. Nevertheless, approximately one third of patients required gastric interposition which is an important consideration when counselling families.

## Data Availability

No datasets were generated or analysed during the current study.
