# Correction: Thoracoscopic staged repair for type A and type B esophageal atresia: outcomes from a tertiary center

**DOI:** 10.1007/s00383-026-06465-9

**Published:** 2026-06-20

**Authors:** Newland Natalia, Snajdauf Jiri, Styblova Jitka, Stepan Coufal, Tereza Bartosova, Rygl Michal, Kokesova Alena

**Affiliations:** 1 Department of Paediatric Surgery, 2nd Faculty of Medicine, Charles University in Prague and Motol University Hospital, Prague, Czech Republic; 2 Laboratory of Cellular and Molecular Immunology, Institute of Microbiology, Czech Academy of Sciences, Prague, Czech Republic

**Correction: Pediatric Surgery International (2026) 42:130** 10.1007/s00383-026-06368-9

In the original version of the article, the following author name Natalia Newland has been printed twice also, the author names have been reversed.

The correct author group should read as follows.

Newland Natalia^1^ · Snajdauf Jiri^1^ · Styblova Jitka^1^ · Stepan Coufal^2^ · Tereza Bartosova^1^ · Rygl Michal^1^ · Kokesova Alena^1^

This has been corrected.

